# 

**Published:** 1890-09-13

**Authors:** 


					.PRICE ONE PENNY.
% Vol. vni-
September 13th, 1890.
^gJBtered at the General Post Office as a Newspaper for
transmission in the United Kingdom and Abroad.]
WITH EXTRA NURSING SUPPLEMENT.
Per Annum, 6s. 6d.j post free.
Monthly Parts, 6<L; post free 8d.
Ditto per Anniun, 8s.. post free.
A WEEKLY JOURNAL OP
Sntmce, /HVftttira, IRttrsmg mtir flbljilantfjropg.
SPECIAL STUDENTS' NUMBER.

				

